# Eucalyptus in Dropsies

**Published:** 1876-10

**Authors:** J. B. Leary


### EUCALYPTUS IN DROPSIES.

BY J. B. LEARY, M. D.

As I intend this paper to be the history of a few cases of general dropsy, in which Eucalyptus was employed, I will not speak of its botany other than to say that according to Professor Von Mueller, there are one hundred and thirty different species of this tree ; and of these I have chosen Eucalyptus Globulus, and the preparation the fluid extract, to be the subject of my paper. It is now nearly four years since I first prescribed Eucalyptus as a specific in gonorrhœa, and it was while treating this disease that I first noticed its remarkable diuretic properties, "the amount of urine passed by some patients while taking it being enormous." I then thought since this causes such an abnormal activity of the renal organs, would it not be advisable to give it in cases of dropsy, and waited an opportunity to verify my suspicions.

The first case in which I tried it was a gentlemen, Mr. R., a resident of Jersey City, who had been told that he had acute

Bright's Disease, and was given but a few weeks to live. I had but little hope of helping him until I saw him the following week, when his condition was so much improved I was led to continue its use; and in seven weeks had the satisfaction of having him go about and assume his usual avocation (packing-box maker.) This patient, when first seen, was unable to lie in the recumbent posture, his limbs were swollen past the capacity of his pantaloons, and he suffered considerable from dyspnœa. I gave him the fluid extract in doses of ten minims four times a day. I should state that, on examination, I found a small quantity of albumen in the urine, but found the liver enlarged and hobnailed, also cardic insufficiency. First saw the patient December, 1874; up to date has no return of dropsy; discontinued Eucalyptus six months ago.

Second Case.—Mrs. McC., aged forty-nine, widow, occupation, housewife, first noticed she had dropsy in 1872; had been tapped three times before I saw her, December, 1874, and each time two gallons of water had been drawn off.

I was called, as I have said, December, 1874, and had to tap to relieve dyspnœa; obtained about half pail of liquid. After tapping, placed her under Eucalyptus and digitalis, as her dropsy was due to cardiac hypertrophy by dilatation; has never had any return of dropsy; still continues taking the remedies first prescribed and enjoys very good health.

Third Case.—Mr. Wm. D., aged thirty-six, occupation none. When first seen, February, 1875, had but been three months discharged from the army; was then under treatment, and his physician, homœpath, had given him up to die. I refused to give him anything, as I thought he had but a few hours to live; but at his own urgent solicitation gave him something, I prescribed digitalis and left. The following day I found him easier, and added Eucalyptus to his digitalis. For four days he remained in "statu quo," and on the fifth day he remarked his legs, which were very much swollen, did not hurt him, and he thought they were getting smaller. That day his left calf was 21 inches in diameter, his right 23, both his thighs measused 33 inches. Fourteen days after his calves were 14 and 16

respectively, his thighs 26. They continued to diminish, until, five weeks after taking his first dose, his calves measured, left 10, right 11; he was able to get on his shoes, and was walking about. This patient gave a specific history. Advised him to stop Eucalyptus, but continue digitalis, as he had slight murmurs. Five months after was called to see him again. His condition was not quite so bad, but his testicles were very much enlarged and painful; did not tap him then, but again placed him under Eucalyptus, and he got well, and has continued taking it. Cause of dropsy is his cardiac disease.

FOURTH CASE.—Mr. J., when first seen, had general anasarca, but not to such a great extent as previous cases. Was placed under Eucalyptus for seven weeks, when he discontinued all medication, being in perfect health, with the exception of cardiac hypertrophy, which does not trouble him.

The fluid extract of Eucalyptus Globulus was given in these cases in doses of ten minims, and never increased, but in some dimished to eight minims, the system at no time tolerating it; and in case three, it acts fully as well to-day as did the first dose. I have also given it in a great many cases of passive congestion of the kidneys, and always with benefit. In fact, whenever I need a diuretic I prescribe it, and have yet to see the case in which it failed, if the kidneys were not so far diseased as to be inert and lose their functions.

Patients, while taking this drug, would sometimes complain of a very severe congestive headache, accompanied with tinnitus aurium; that their appetite was very much better, though no tonic was prescribed, showing a similarity to quinia, and in some cases, a laxative condition of the bowels was produced.

Some may try this remedy and be disappointed in the result, which I think will be owing to the preparation used, or rather by whom prepared. Some may wish to give it in combination with other diuretics, and will find most preparations to be incompatible, owing to the resin which it contains being precipitated. I have found that prepared by the firm of Lazell, Marsch & Gardner, of New York, to be the best, as it does not precipitate with acids or alkalies.—*King's County Medical Society.*

